# Supplementary material for: Clustering by measuring local direction centrality for data with heterogeneous density and weak connectivity
Source: Nat Commun. 2022 Sep 16;13:5455. doi: 10.1038/s41467-022-33136-9 (PMC9481560; doi:10.1038/s41467-022-33136-9)
Supplement: Supplementary file 1 — Supplementary Information [file 41467_2022_33136_MOESM1_ESM.pdf]

Clustering by measuring local direction centrality for data with heterogeneous density and weak connectivity

Dehua Peng[1, 2, 3], Zhipeng Gui[2, 3, 4, *], Dehe Wang[5, 6], Yuncheng Ma[2, 3], Zichen Huang[2, 3], Yu Zhou[5, 6], Huayi Wu[1, 3, 4]

Author Affiliations:

[1] State Key Laboratory of Information Engineering in Surveying, Mapping and Remote Sensing, Wuhan University, Wuhan, China.

[2] School of Remote Sensing and Information Engineering, Wuhan University, Wuhan, China.

[3] Collaborative Innovation Center of Geospatial Technology, Wuhan University, Wuhan, China.

[4] Hubei Luojia Laboratory, Wuhan, China

[5] State Key Laboratory of Virology, Modern Virology Research Center, College of Life Sciences, Wuhan University, Wuhan, China.

[6] Frontier Science Center for Immunology and Metabolism, Wuhan University, Wuhan, China.

Supplementary Note 1: Normalization of DCM in 2D space

By measuring the variance of all angles formed by the neighborhood points, we define the Direction Centrality Metric (*DCM*) as follows:

$$DCM = \frac{1}{k}\sum_{i=1}^{k}\left(\alpha_i - \frac{2\pi}{k}\right)^2$$

where $\sum_{i=1}^{k}\alpha_i = 2\pi$ $(\alpha_1, \alpha_2 \dots \alpha_k > 0)$, k denotes the number of nearest neighbors, α_i refers to the angle formed by the adjacent neighbors and center point, if and only if $\alpha_1 = \alpha_2 = \cdots = \alpha_k$, $\min(DCM) = 0$. Meanwhile, the maximum of DCM can be solved by the following derivation:

$$\begin{aligned}
DCM = \frac{1}{k}\sum_{i=1}^{k}\left(\alpha_i - \frac{2\pi}{k}\right)^2 &= \frac{1}{k}\sum_{i=1}^{k}\left({\alpha_i}^2 - \frac{4\pi}{k}\alpha_i + \frac{4\pi^2}{k^2}\right) \\
&= \left(\frac{1}{k}\sum_{i=1}^{k}{\alpha_i}^2\right) - \frac{4\pi}{k^2}\sum_{i=1}^{k}\alpha_i + \frac{4\pi^2}{k^2} = \left(\frac{1}{k}\sum_{i=1}^{k}{\alpha_i}^2\right) - \frac{4\pi^2}{k^2} \\
&\le \frac{1}{k}\left(\sum_{i=1}^{k}\alpha_i\right)^2 - \frac{4\pi^2}{k^2} \le \frac{4\pi^2}{k} - \frac{4\pi^2}{k^2} = \frac{4(k-1)\pi^2}{k^2}
\end{aligned}$$

When one of the angles is equal to 2π and the remaining angles are 0, $\max(DCM) = \frac{4(k-1)\pi^2}{k^2}$. According to the minimum and maximum of DCM, we can normalize DCM to the range [0, 1] as follows:

$$\begin{aligned}
\widehat{DCM} &= \frac{DCM - \min(DCM)}{\max(DCM) - \min(DCM)} \\
&= \frac{k}{4(k-1)\pi^2}\sum_{i=1}^{k}\left(\alpha_i - \frac{2\pi}{k}\right)^2
\end{aligned}$$

Supplementary Note 2: Pseudocode of CDC algorithm and the adaptive method to determine T_{DCM} in 2D space

Algorithm 1 Clustering by Measuring Local Direction Centrality (CDC)

Input: the dataset $X(x_1, x_2 \dots x_n)$, k for KNN and DCM threshold T_{DCM}

for each point x_i
Search the k nearest neighbors of point x_i;
Calculate the k angles formed by its KNNs;
Calculate the normalized DCM_i of point x_i;
if $DCM_i > T_{DCM}$
Add the point x_i to the set of boundary points B;
else
Add the point x_i to the set of internal points I;
end if
end for
for each internal point x_i in I
Calculate the distances between x_i and all the points in B;
Select the minimum as the reachable distance r_i of x_i;
end for
Initialize the point labels $C(c_1, c_2 \dots c_n)$ as a zero vector (i.e., unlabeled);
Set $temp = 1$;
for each internal point x_i in I and $c_i == 0$
for another internal point x_j in I
$c_i = temp$;
Calculate the distance d_{ij} between x_i and x_j;
if $d_{ij} \le r_i + r_j$ and $c_j == 0$
$c_j = c_i$;
else if $d_{ij} \le r_i + r_j$ and $c_j > 0$
Assign c_i to all points whose labels equal to c_j;
end if
end for
$temp = temp + 1$;
end for
for each boundary point x_i in B
Assign the label of the nearest internal point to x_i;
end for
return the cluster labels C;

Algorithm 2 Adaptive Method for T_{DCM} in 2D space

Input: the dataset $X(x_1, x_2 \dots x_V)$, k for KNN, the number of clusters C

for each point x_i
    Search the k nearest neighbors of point x_i;
    Calculate the k angles formed by its KNNs;
    Calculate the normalized DCM_i of point x_i;
end for
Sort the *DCMs* in a descend order as $DCM_1, DCM_2, \dots, DCM_V$;
Construct the initial TIN using Delaunay algorithm;
Count the number of triangles F in the TIN;
for each triangle $\mathcal{T}$
    if $\mathcal{T}$ is a cross-cluster triangle
        $F = F - 1$;
    end if
end for
Calculate the number of boundary points $B = 2V - F - 2C$;
return DCM_B;

Supplementary Note 3: Evaluation metrics of clustering performance

To evaluate the clustering performance quantitatively, we adopt four validity indexes Accuracy (ACC), Normalized Mutual Information (NMI), Adjusted Rand Index (ARI), F1-score.

ACC refers to the accuracy rate of the clustering results compared with the true labels. We set the true label vector and the predicted label vector as $\boldsymbol{l} = (l_1, l_2, \dots, l_n) \in \mathbb{R}^n$ and $\boldsymbol{r} = (r_1, r_2, \dots, r_n) \in \mathbb{R}^n$ respectively, and the ACC can be defined as:

$$\text{ACC} = \frac{\sum_{i=1}^{n} \delta\big(l_i, map(r_i)\big)}{n}$$

where $\delta(\cdot)$ denotes an indicator function:

$$\delta(x, y) = \begin{cases} 1 & \text{if} \quad x = y \\ 0 & \text{otherwise} \end{cases}$$

$map(\cdot)$ is a mapping function that maps each predicted label to one of the true cluster label. Commonly, the best mapping can be found by using the Kuhn-Munkres or Hungarian Algorithm.

NMI measures the agreement of predict and true assignments, ignoring permutations. It can be defined as:

$$\text{NMI} = \frac{\sum_{i=1}^{|L|} \sum_{j=1}^{|R|} \left|L_i \cap R_j\right| \log \dfrac{n\left|L_i \cap R_j\right|}{|L_i|\left|R_j\right|}}{\sqrt{\left(\sum_{i=1}^{|L|} |L_i| \log \dfrac{|L_i|}{n}\right)\left(\sum_{j=1}^{|R|} |R_j| \log \dfrac{|R_j|}{n}\right)}}$$

where L_i denote the point set of the ith cluster that predicted by the algorithm, while R_j denotes the jth cluster of the true labels.

We define the number of point pairs that belong to the same clusters in both of the true and predicted labels as TP, that belong to the different clusters in both of the true and predicted labels as TN, that belong to the same clusters in the true labels but not in the predicted labels as FP, that belong to the same clusters in the predicted labels but not in the true labels as FN. Then we have:

$$\text{ARI} = \frac{2(TP \cdot TN - FP \cdot FN)}{2(TP \cdot TN - FP \cdot FN) + (FP + FN)(TP + TN + FP + FN)}$$

$$\text{F1-score} = \frac{2TP}{2TP + FP + FN}$$

Supplementary Note 4: Preprocessing and parameter settings for clustering on scRNA-seq datasets

We selected seven biological clustering pipelines and seven classical clustering methods for comparing with CDC on scRNA-seq datasets. The preprocessing and clustering of all scRNA-seq datasets were conducted on a commodity desktop computer with a 8-core Intel i7 processor and 64 GB RAM, except for MIHPF which was preprocessed on an Inspur Tiansuo Service with total 176 cores and 4 TB DDR4 memory.

<u>*Biological clustering methods*</u>

1) For Seurat method, the filtered UMI (Unique Molecular Identifiers) matrix of published scRNA-seq data was preprocessed with *NormalizeData*, *FindVariableFeatures*, *ScaleData* and *RunPCA* functions in Seurat package (v3.2.2) with default parameter firstly. The first 50 principal components of PCA were selected. A shared nearest-neighbor (SNN) graph was constructed and clusters were found by *FindClusters* function. The detailed settings of the parameters were as follows:

Function	Parameter	Parameter space
FindNeighbors	dim	5~50
FindClusters	resolution	0.1~1.0 (interval = 0.1)

2) For monocle3 method (v0.2.1), the original filtered UMI matrix was processed with *preprocess_cds* function firstly, and the UMAP method was used for dimension reduction. Clusters were predicted with *cluster_cells* function in UMAP space. The detailed settings of the parameters were as follows:

Function	Parameter	Parameter space
preprocess_cds	num_dim	50
reduce_dimension	reduction_method	UMAP
reduce_dimension	umap.n_neighbors	5, 10, 15, 20, 25, 30
reduce_dimension	umap.min_dist	0.01, 0.02, 0.05, 0.1, 0.2, 0.3, 0.4, 0.5, 0.6, 0.7, 0.8, 0.9
cluster_cells	k	5~50 (interval = 5)
cluster_cells	num_iter	5

3) For SC3 method, the original filtered UMI matrix was used as input data. After PCA reduction, all steps of SC3 analysis were executed by sc3 method (v1.14.0) with parameter ks = 2~50.

4) For the MetaCell method, a single cell database was created by *scdb_init* function firstly. Then the original UMI matrix was added to this database using *scdb_add_mat* function. No expressed genes and low expressed cells (i.e., UMI<800) were removed using *mcell_mat_ignore_genes* and *mcell_mat_ignore_small_cells* functions respectively. After preprocessing with *mcell_add_gene_stat* function, the *mcell_gset_filter_varmean* function is used to find HVG (Highly Variable Genes) with recommended parameters T_vm = 0.2. Finally, we adopted three functions, i.e., *mcell_add_cgraph_from_mat_bknn*, *mcell_coclust_from_graph_resamp* and *mcell_mc_from_coclust_balanced* in sequence for clustering. The detailed settings of the parameters were as follows:

**Function**	**Parameter**	**Parameter space**
mcell_add_cgraph_from_mat_bknn	k	50, 75, 100, 150, 200
mcell_add_cgraph_from_mat_bknn	dsamp	TRUE
mcell_coclust_from_graph_resamp	min_mc_size	20
mcell_coclust_from_graph_resamp	p_resamp	0.75, 0.9, 0.95
mcell_coclust_from_graph_resamp	n_resamp	500
mcell_mc_from_coclust_balanced	k	25, 30 ,35, 40, 45, 50
mcell_mc_from_coclust_balanced	min_mc_size	30
mcell_mc_from_coclust_balanced	alpha	1, 1.5, 2

5) For dropClust method (v2.1.0), the original filtered UMI matrix was used as input data. Genes with UMI greater than 3 in a cell are called expressed. UMI matrix with cells with more than 3 expressed genes and genes with more than 3 expressed cells was used in the further analysis. The expressed UMI matrix was normolized by *CountNormalize* function and highly variable genes were selected using *RankGenes* function. Then, *Cluster* function was used to predict the clusters. The detailed settings of the parameters were as follows:

**Function**	**Parameter**	**Parameter space**
Cluster	method	“louvian”, “hclust”, “kmeans”
Cluster	k_nn	5, 10, 15, 20, 25, 30, 35, 40
Cluster	conf	0.01, 0.02, 0.05, 0.1, 0.2, 0.3, 0.4, 0.5, 0.6, 0.7, 0.75, 0.8, 0.9, 0.95

6) For two SNN-based (Shared Nearest-Neighbor) graph partition methods, i.e., SNN-Louvain and SNN-Walktrap, the nearest-neighbor graph was constructed with *buildSNNGraph* function in scran package (v1.14.6) based on the dimensional reduction data. Then *cluster_walktrap* and *cluster_louvain* function in igraph package (v1.2.6) and *mcl* function in MCL package (v1.0) were

used to predict the clusters. MCL algorithm was not evaluated in the final results due to the overlong computing time. The detailed settings of the parameters were as follows:

Function	Parameter	Parameter space
buildSNNGraph	use.dimred	UMAP: 2<br>PCA: 2, 3, 4, 5, 10, 15, 20, 25, 30, 35, 40, 45, 50
buildSNNGraph	k	2, 3, 4, 5, 6, 7, 8, 9, 10, 15, 20, 25, 30
buildSNNGraph	type	"rank", "number", "jaccard"
cluster_walktrap	steps	1~10
mcl	addLoops	TRUE
mcl	inflation	1, 1.5, 2, 2.5, 3
mcl	expansion	1.5, 1.75, 2, 2.25, 2.5

Classical clustering methods

AGNES (cluster::agnes, v2.1.2), DIANA (cluster::diana, v2.1.2), Hierarchical Clustering (stats::hclust, v3.6.1), DBCSAN (dbscan::dbscan, v1.1.8), K-means (stats::kmeans, v3.6.1), C-means (e1071::cmeans, v1.7.3), and CLARA (cluster::clara, v2.1.2) were used to predict the clusters based on the data after dimension reduction (2 dimension for UMAP and 2, 3, 4, 5, 10, 15, 20, 25, 30, 35, 40, 45, 50 for PCA). UMAP and PCA were conducted on the preprocessed data by Seurat. The detailed settings of the parameters were as follows:

Function	Parameter	Parameter space
agnes	metric	"euclidean", "manhattan"
	method	"average", "single", "complete", "ward"
	k	2~50
diana	metric	"euclidean", "manhattan"
	k	2~50
hclust	metric	"euclidean", "manhattan", "maximum", "canberra", "binary", "minkowski"
	method	"ward.D", "ward.D2", "single", "complete", "average", "mcquitty", "median", "centroid"
	k	2~50
dbscan	eps	0.02, 0.05, 0.1, 0.2, 0.3, 0.4, 0.5, 0.6, 0.7, 0.8, 0.9, 1, 2
	minPts	3, 4, 5, 6, 7, 8, 9, 10, 15, 20, 25, 30
kmeans	k	2~50
cmeans	k	2~50
clara	metric	"euclidean", "manhattan", "jaccard"
	k	2~50

CDC method

We preprocessed the raw scRNA-seq datasets using Seurat pipeline, and adopted *RunPCA* function to reduce the dimensions to 50 on the scaled data. Then, UMAP was used to embed 50 PCs to 2D-

5D spaces. We walked through the combinations of *n_neighbors* and *min_dist* in UMAP and selected the best results to present in Fig. 2. We vary *ratio* of CDC in the range of 0.85 to 0.99 on 12 scRNA-seq datasets with an interval of 0.01, and in a smaller range of 0.95 to 0.99 for dataset AMB with an interval of 0.005. Since the cell types of AMB are composed of multiple separated parts, less boundary points are required to connect the parts into a whole cluster. The detailed settings of the parameters were as follows:

Function	Parameter	Parameter space
umap	n_neighbors	5~40 (interval = 5)
	min_dist	0.1~1.0 (interval = 0.1)
	n_components	2, 3, 4, 5
CDC	k	30, 40, 50
	ratio	BH, BM, Muraro, Segerstolpe, Xin, ALM, VISp, TM, WT_R1, WT_R2, NdpKO_R1, NdpKO_R1: 0.85~0.99 (interval = 0.01)
		AMB: 0.95~0.99 (interval = 0.005)

Preprocessing and parameter settings on MIHPF

The entire preprocessing pipeline was performed on the supercomputing center, while the clustering process was conducted on a commodity desktop computer. The supercomputing center provided us an Inspur Tiansuo Service that has 8 Intel(R) Xeon(R) CPU E7-8880 v4 with 176 cores, 4TB DDR4 memory, 500GB SSD and 8TB HDD. We firstly removed 4,014 cells without labels of class and subclass before clustering. Then, MIHPF was partitioned into multiple sub-datasets according to the donor IDs. Each sub-dataset was preprocessed using Seurat v3. PCA was used to extract the first 50 principal components and UMAP conducts dimension reduction based on the PCA space. SNN-Louvain adopts *buildSNNGraph* function to build the SNN-based graph. The detailed settings of the parameters were as follows:

Function	Parameter	Parameter space
umap	n_neighbors	50
	n_components	2
kmeans	k	30~50
buildSNNGraph	k	10~60
CDC	k	30~50
	ratio	0.7~0.99

Supplementary Note 5: Preprocessing and parameter settings for clustering on CyTOF datasets

The preprocessed CyTOF datasets were downloaded from FlowRepository (repository FR-FCM-ZZPH) (Spidlen et al., 2012). As described in (Weber et al., 2016), "the data pre-processing included the application of an *arcsinh* transformation with a standard cofactor of 5 (CyTOF data) or 150 (flow cytometry data). For the flow cytometry datasets, pre-gating to exclude doublets, debris, and dead cells was also required. The clustering algorithms were run on all remaining single, live cells and no additional pre-gating was performed, since our aim is to evaluate performance in maximally automated settings. In addition, we did not perform any standardization of individual protein marker dimensions. This was unnecessary since the *arcsinh* already transforms all dimensions to comparable scales. More importantly, standardization of dimensions that do not contain a true signal could amplify the effect of noise and outliers, adversely affecting clustering performance."

We performed clustering on the cells after removing the ones without manually gated population labels, and the ARI score was subsequently calculated on the subset of known cells to evaluate the clustering accuracy. We followed the parameter settings in (Weber et al., 2016) and the default settings in software (ACCENSE) or online platforms (FLOCK). We specified the number of clusters for six algorithms (flowClust, flowMeans, flowMerge, flowSOM, K-means and Rclusterpp) according to the number of manually gated populations (C = 14 on Levine, C = 24 on Samusik), which was set as 40 in the original paper. All the clustering algorithms were implemented on a commodity desktop computer with an 8-core Intel i7 processor and 64 GB RAM. The recorded running times include the processes of clustering and dimensionality reduction. Due to the computability issue, the running times of ACCENSE, DensVM and flowMerge are counted on the subsampling datasets. The detailed parameter settings are presented as follows:

**Method (Environment)**	**Dataset**	**Parameter settings**	**No. of cells**
ACCENSE (ACCENSE 0.5.1)	Levine	z-score = TRUE, remove outliers = TRUE<br>dimensionality reduction = Barnes-Hut-SNE<br>no_dims = 2, perplexity = 30, theta = 0.5	All
	Samusik	as above	400,000
clusterX (R v4.0.3)	Levine	cytof_dimReduction: method = "tsne"	All
	Samusik	as above	All

DensVM (R v4.0.3)	Levine	cytof_dimReduction: method = “tsne”	All
	Samusik	as above	300,000
FLOCK (ImmPort Galaxy)	Levine	bins = 6, density = 3	All
	Samusik	as above	All
flowClust (R v4.0.3)	Levine	C = 14	All
	Samusik	C = 24	All
flowMeans (R v4.0.3)	Levine	Standardize = FALSE, C = 14	All
	Samusik	Standardize = FALSE, C = 24	All
flowMerge (R v4.0.3)	Levine	NA	NA
	Samusik	C = 24	10,000
flowPeaks (R v4.0.3)	Levine	all defaults	All
	Samusik	as above	All
flowSOM (R v4.0.3)	Levine	Scaled = FALSE, GridSize = 10, C = 14	All
	Samusik	Scaled = TRUE, GridSize = 10, C = 24	All
immunoClust (R v4.0.3)	Levine	classify.all = TRUE	All
	Samusik	as above	All
K-means (Matlab R2020b)	Levine	C = 14	All
	Samusik	C = 24	All
MeanShift (Matlab R2020b)	Levine	bandwidth = 5	All
	Samusik	bandwidth = 6	All
PhenoGraph (Python v3.7)	Levine	k = 30, metric = “euclidean”	All
	Samusik	as above	All
Rclusterpp (R v4.0.3)	Levine	C = 14, method = “ward”	All
	Samusik	C = 24, method = “ward”	All
SamSPECTRAL (R v4.0.3)	Levine	normal.sigma = 100, separation.factor = 1	All
	Samusik	as above	All
CDC-U2 (UMAP: Java v15.0.1 CDC: Matlab R2020b)	Levine	n_neighbors = 50, n_components = 2, k = 60, ratio = 0.9667	All
	Samusik	n_neighbors = 30, n_components = 2, k = 27, ratio = 0.9750	All

References:

Spidlen, J., Breuer, K., Rosenberg, C., Kotecha, N. & Brinkman, R. R. FlowRepository: A resource of annotated flow cytometry datasets associated with peer-reviewed publications. Cytom. Part A 81A, 727-731 (2012).

Weber, L. M. & Robinson, M. D. Comparison of Clustering Methods for High-Dimensional Single-Cell Flow and Mass Cytometry Data. Cytom. Part A 89, 1084-1096 (2016).

Supplementary Note 6: Calculation of the volume of parallelepiped and simplex in high-dimensional space

High-dimensional parallelepiped volume calculation

We define the k-dimensional parallelepiped P in vector space $\mathbb{R}^d$ is determined by vectors $\boldsymbol{\varepsilon}_1$, $\boldsymbol{\varepsilon}_2$, …, $\boldsymbol{\varepsilon}_k$ where $k \leq d$. Take the span of these vectors with t_i and let $P = P(\boldsymbol{\varepsilon}_1, \boldsymbol{\varepsilon}_2, \ldots, \boldsymbol{\varepsilon}_k) = \{t_1\boldsymbol{\varepsilon}_1 + t_2\boldsymbol{\varepsilon}_2 + \cdots + t_k\boldsymbol{\varepsilon}_k | 0 \leq t_i \leq 1\}$. Using the Gram-Schmidt process, we can find a set of orthonormal bases $\boldsymbol{e}_1$, $\boldsymbol{e}_2$, …, $\boldsymbol{e}_k$ that satisfies

$$(\boldsymbol{\varepsilon}_1, \boldsymbol{\varepsilon}_2, \ldots, \boldsymbol{\varepsilon}_k) = (\boldsymbol{e}_1, \boldsymbol{e}_2, \ldots, \boldsymbol{e}_k)Q$$

where $Q = (q_{ij})$ is the transition matrix and $\varepsilon_j = q_{1j}\boldsymbol{e}_1 + q_{2j}\boldsymbol{e}_2 + \cdots + q_{ij}\boldsymbol{e}_i$. Actually, $\boldsymbol{e}_k$ is orthogonal with $\boldsymbol{\varepsilon}_1$, $\boldsymbol{\varepsilon}_2$, …, $\boldsymbol{\varepsilon}_{k-1}$ and the projection of $\boldsymbol{\varepsilon}_k$ onto $\boldsymbol{e}_k$ is q_{kk}. Since the volume is equal to the product of base area and the height, we have

$$vol\big(P(\boldsymbol{\varepsilon}_1, \boldsymbol{\varepsilon}_2, \ldots, \boldsymbol{\varepsilon}_k)\big) = vol\big(P(\boldsymbol{\varepsilon}_1, \boldsymbol{\varepsilon}_2, \ldots, \boldsymbol{\varepsilon}_{k-1})\big)q_{kk}$$

The volume of P can be calculated recursively by

$$vol(P) = q_{11}q_{22} \ldots q_{kk} = \det(Q)$$

Let $A = (\boldsymbol{\varepsilon}_1, \boldsymbol{\varepsilon}_2, \ldots, \boldsymbol{\varepsilon}_k)^T$, its Gram matrix is

$$G(\boldsymbol{\varepsilon}_1, \boldsymbol{\varepsilon}_2, \ldots, \boldsymbol{\varepsilon}_k) = AA^T = Q^T(\boldsymbol{e}_1, \boldsymbol{e}_2, \ldots, \boldsymbol{e}_k)^T(\boldsymbol{e}_1, \boldsymbol{e}_2, \ldots, \boldsymbol{e}_k)Q = Q^TQ$$

Thus, the volume of P can be rewritten as:

$$vol(P) = \sqrt{\det(\boldsymbol{A}\boldsymbol{A}^T)}$$

Simplex volume calculation

To better understand the calculation of the high-dimensional simplex volume, we give three intuitive examples of 2-simplex s_2, 3-simplex s_3 and 4-simplex s_4 in Fig. S1. Differentiation process is adopted to calculate the simplex volume. In 2D space, the volume of s_2 can be expressed in integrated form:

$$vol(s_2) = \int_0^{h_1} s \; dh$$

Considering the same ratio of corresponding heights and bases in s_2, we have

$$\frac{s}{vol(s_1)} = \frac{h}{h_1}$$

Then, the volume of s_2 can be rewritten as:

$$vol(s_2) = \frac{vol(s_1)}{h_1}\int_0^{h_1} h \ \ dh = \frac{1}{2}vol(s_1)h_1$$

Similarly, the volume of s_3 in 3D space can be calculated:

$$vol(s_3) = \int_0^{h_2} s \ \ dh$$

But the ratio of corresponding bases is the square of that of heights, so we have

$$\frac{s}{vol(s_2)} = (\frac{h}{h_2})^2$$

Thus, the volume of s_2 can be rewritten as:

$$vol(s_3) = \frac{vol(s_1)}{{h_2}^2}\int_0^{h_2} h^2 \ \ dh = \frac{1}{3}vol(s_2)h_2$$

In the same way, the volume of 4-simplex s_4 can be calculated

$$vol(s_4) = \frac{vol(s_3)}{{h_3}^3}\int_0^{h_3} h^3 \ \ dh = \frac{1}{4}vol(s_3)h_3$$

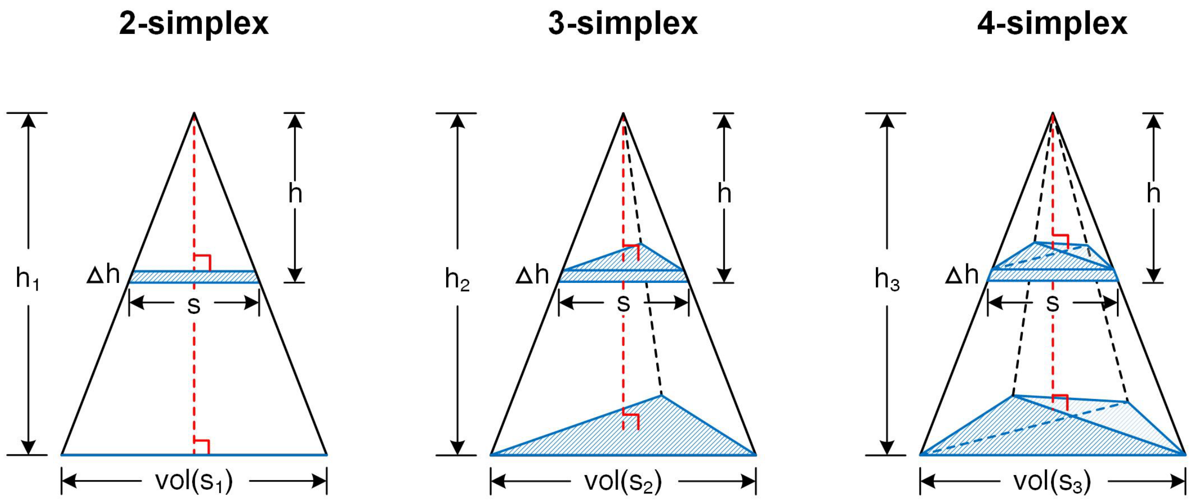

Supplementary Fig. 1. Illustration of simplex volume calculation in space of different dimensions.

Let s_d be the d-simplex, its volume can be generalized as:

$$vol(s_d) = \frac{1}{d}vol(s_{d-1})h_{d-1}$$

It can be calculated recursively as:

$$vol(s_d) = \frac{1}{d} vol(s_{d-1}) h_{d-1} = \frac{1}{d!} vol(s_1) h_{d-1} h_{d-2} \dots h_1 = \frac{1}{d!} \prod_{i=0}^{d-1} h_i$$

Actually, h_0, $h_1, \dots,$ h_{d-1} can determine a d-dimensional parallelepiped P_d. Since they are the edges of P and are orthogonal to each other, the volume of P_d can be calculated

$$vol(P_d) = \prod_{i=0}^{d-1} h_i$$

Therefore, the volume of s_d can be rewritten as:

$$vol(s_d) = \frac{vol(P_d)}{d!} = \frac{vol(P_d)}{\Gamma(d)}$$

Supplementary Note 7: Evaluating the parameter sensitivity via stratified sampling and random perturbation

Sensitivity analysis aims to identify parameters that do or do not have a significant influence on simulation models of real-world observations. We evaluated the sensitivity of the two input parameters of CDC, i.e., k and T_{DCM}, via stratified sampling and random perturbation (Fig. S2). Specifically, we firstly divided the value range of the two parameters using 10 equal intervals respectively, which generated a 10 by 10 grid with cells of the same size. Then we randomly selected one sampling point in each cell and exert a positive or negative perturbation to each point. We specified five fixed perturbations for each parameter. Only one parameter was varied at a time and the other was fixed. Finally, we calculated the sensitivity indexes that measures the relative change of the clustering quality before and after perturbations. Each group of experiment was conducted five times to avoid randomness in the simulations.

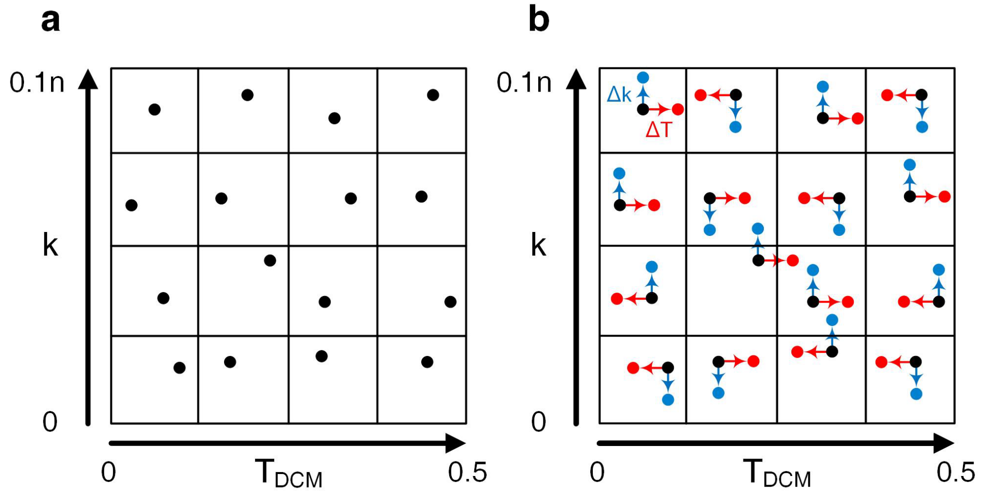

Supplementary Fig. 2. Illustration of sensitivity analysis using stratified sampling and random perturbation. (**a**) Sampling points generated by stratified sampling. (**b**) Operating random perturbations that could be either positive or negative to each sampling point.

Adjusted Rand Index (ARI) was used as the dependent variable, since it is sensitive to the clustering quality. To ensure that ARI is positive ranging from 0 to 1, we conducted the sampling in the range of k from 0 to $0.1n$ and that of T_{DCM} from 0 to 0.5. Because the clustering qualities obtained by most of sampling points make no sense when k is greater than $0.1n$ or T_{DCM} surpass 0.5 (e.g., all the points are identified as internal points that form a single cluster), which

would generate excess invalid results (ARI < 0) that affect the effectiveness of the sensitivity evulation. The sensitivity indexes S_k, S_T of k and T_{DCM} are defined as:

$$S_k = \frac{1}{100}\sum_{i=1}^{100}\left|\frac{\mathrm{ARI}(k_i + \Delta k, T_i) - \mathrm{ARI}(k_i, T_i)}{\mathrm{ARI}(k_i + \Delta k, T_i) + \mathrm{ARI}(k_i, T_i)}\right|$$

$$S_T = \frac{1}{100}\sum_{i=1}^{100}\left|\frac{\mathrm{ARI}(k_i, T_i + \Delta T) - \mathrm{ARI}(k_i, T_i)}{\mathrm{ARI}(k_i, T_i + \Delta T) + \mathrm{ARI}(k_i, T_i)}\right|$$

Theoretically, almost all ARIs range from 0 to 1. In this case, the two sensitivity indexes are also between 0 and 1. Hence, we divide their value range evenly into four intervals and each interval indicates one sensitivity level (i.e., Insensitive: 0-0.25, Mild sensitive: 0.25-0.5, Sensitive: 0.5-0.75, Hypersensitive: 0.75-1.00). The result is presented in Table S6.

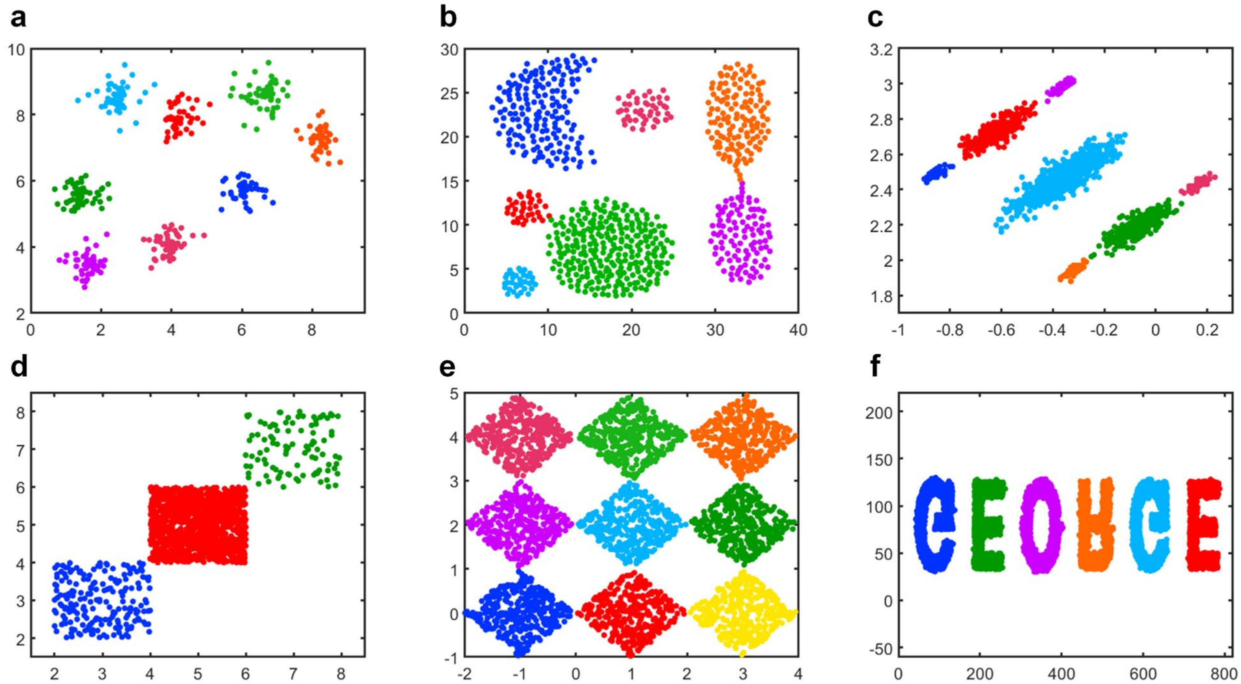

Supplementary Fig. 3. Clustering results of CDC on six synthetic datasets (DS4-DS9), with (a) 422, (b) 788, (c) 1,000, (d) 1,500, (e) 2,000, (f) 6,847 points respectively.

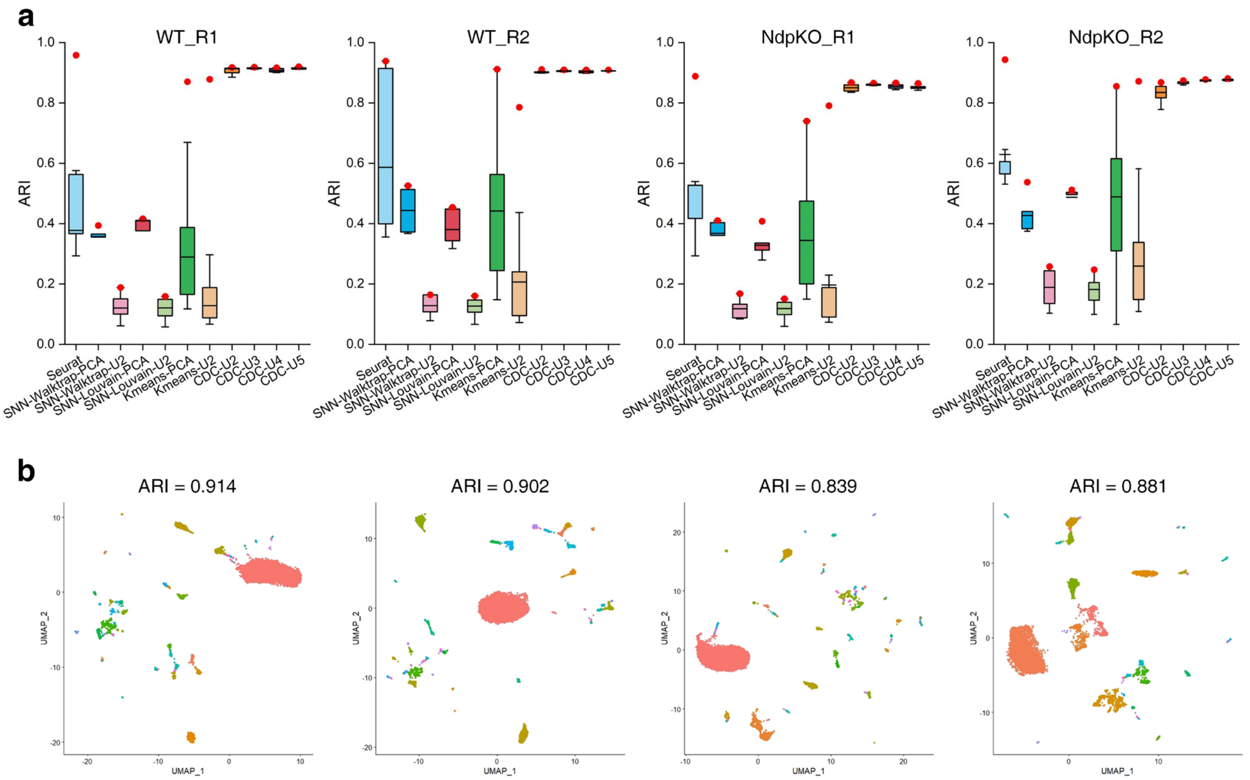

Supplementary Fig. 4. Clustering performances on four mouse retina datasets. (**a**) Clustering accuracies reported by ARI score of five algorithms conducted in PCA and UMAP spaces. Boxes show the median and the 25-75% range, while whiskers refer to the 1.5 times interquartile range. (**b**) Clustering results of CDC with default parameter settings (*n_components* = 2, *k* = 30, *ratio* = 0.9). CDC achieved comparable max ARI score with Seurat and the most stable outcomes among all the methods. It can also obtain promising clustering results using the default parameter settings.

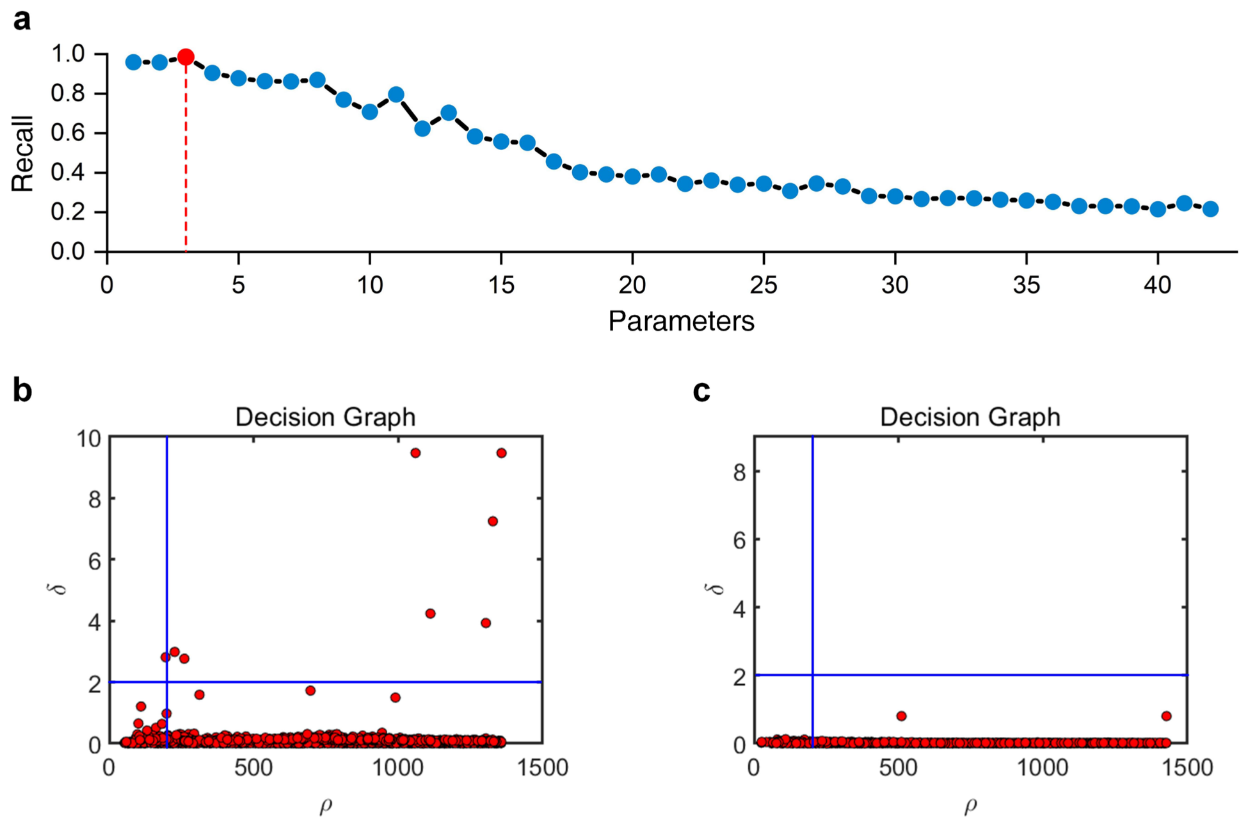

Supplementary Fig. 5. Criterion for conducting multi-round clustering optimization. (**a**) Selecting the clusters with high recall score as initial result. (**b**) Conducting the next round of clustering if there are multiple significant density peaks ($\rho > 200$, $\delta > 2$) in the decision graph. (**c**) Clustering terminates if there is no density peak in the decision graph.

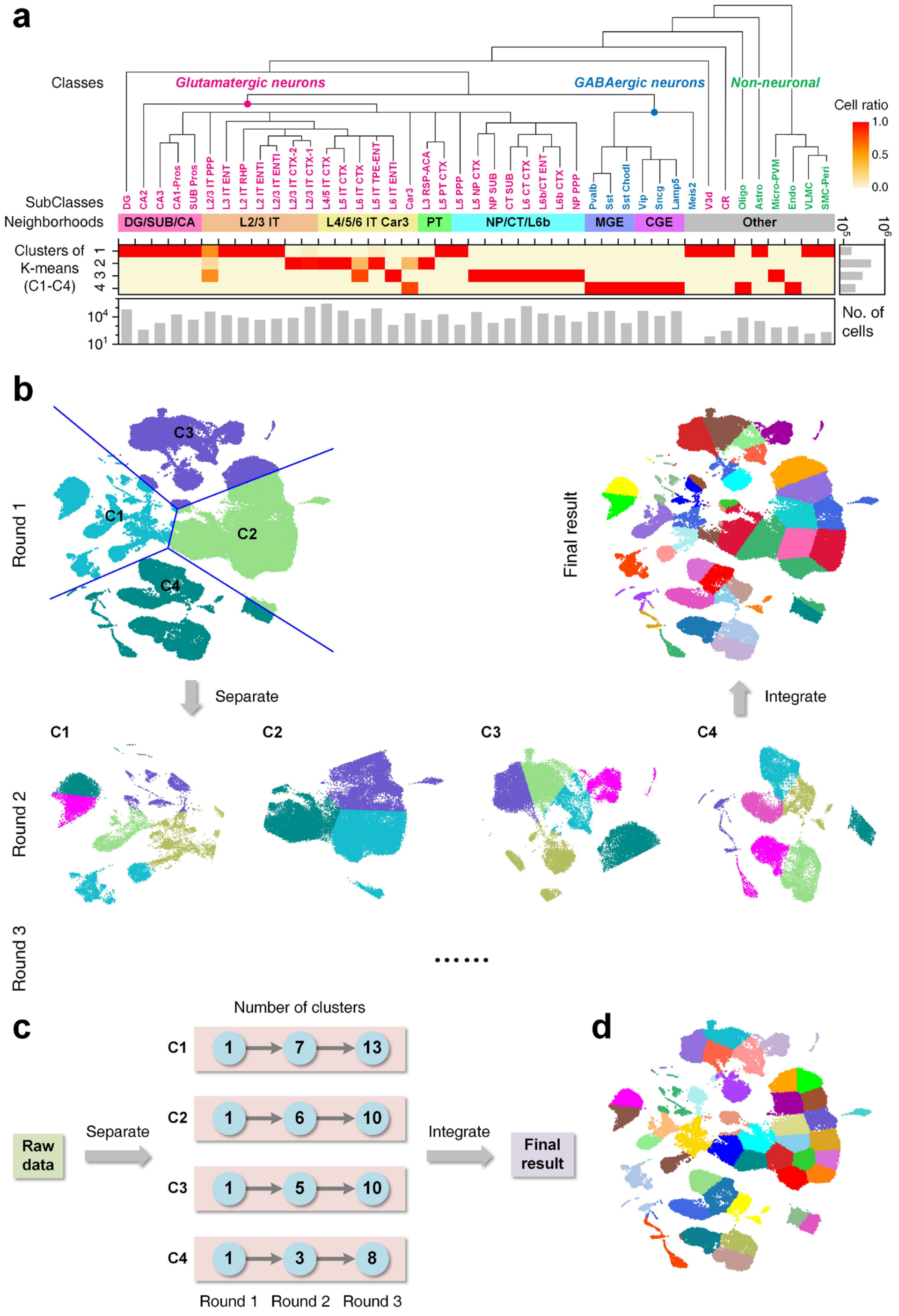

Supplementary Fig. 6. Multi-round K-means clustering on MIHPF dataset. (**a**) Confusion matrix of the K-means results having the highest recall score (C = 4). (**b**) Clustering results of K-means in 2D UMAP space through three rounds of clustering optimization. It reached the highest ARI in the second round. (**c**) A network indicated the number of clusters setting in each round of K-means clustering. (**d**) Clustering result of K-means when setting the number of clusters as 43 directly.

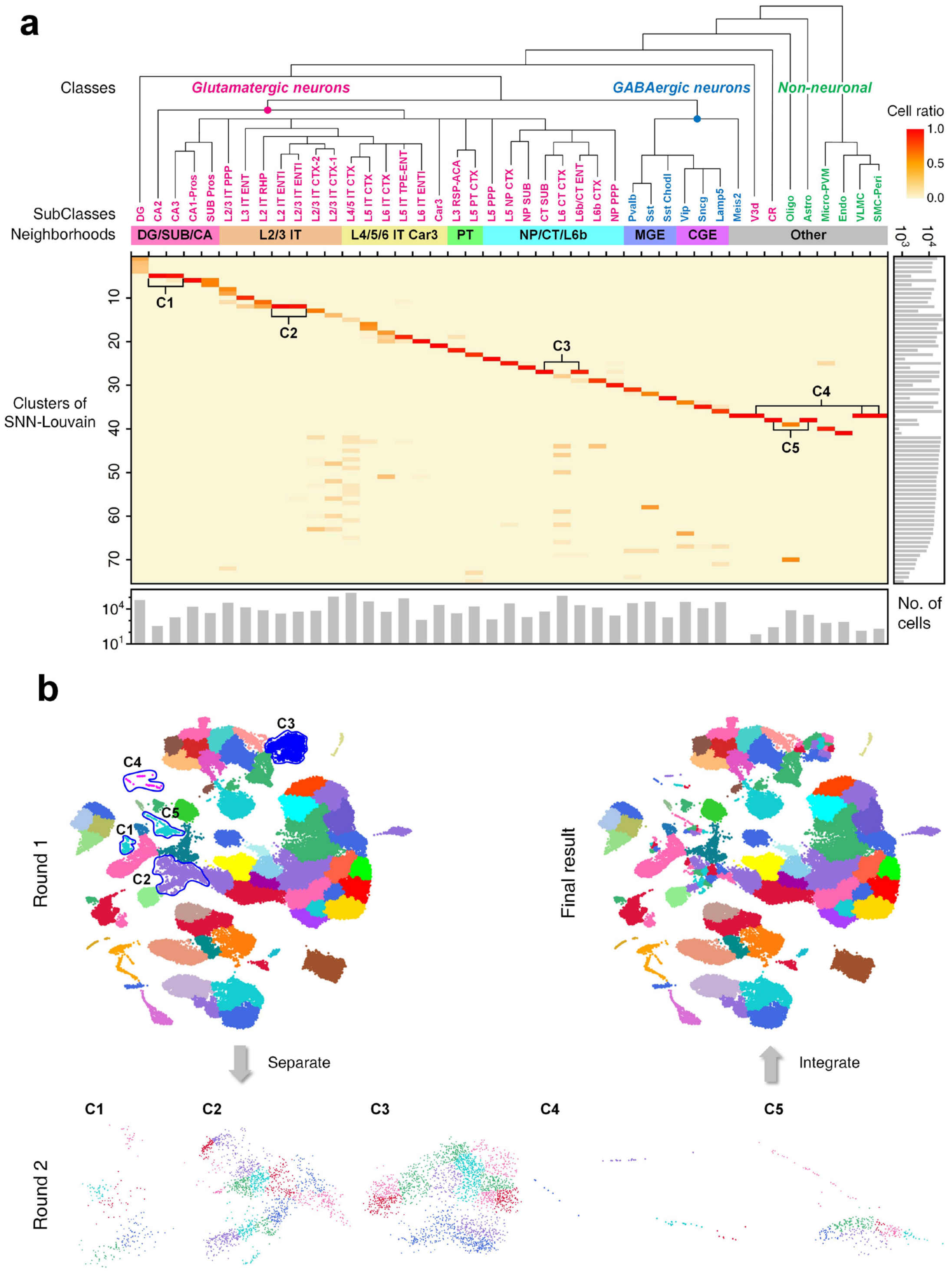

Supplementary Fig. 7. Multi-round SNN-Louvain clustering on MIHPF dataset. (a) Confusion matrix of the SNN-Louvain result having the highest recall score (k = 48). **(b)** Clustering results of SNN-Louvain in 2D UMAP space through two rounds of clustering optimization and the clustering accuracy has not been improved.

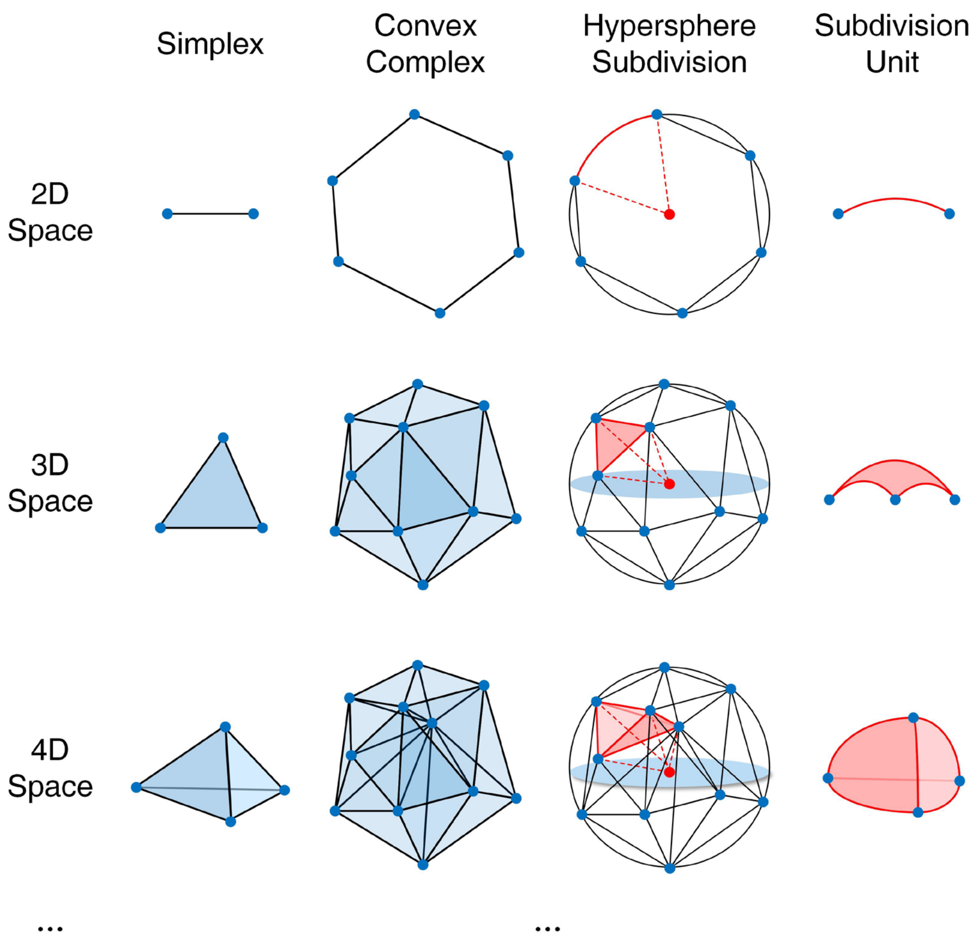

Supplementary Fig. 8. Graphical illustration of expanding DCM with hyperspherical subdivisions for high-dimensional spaces.

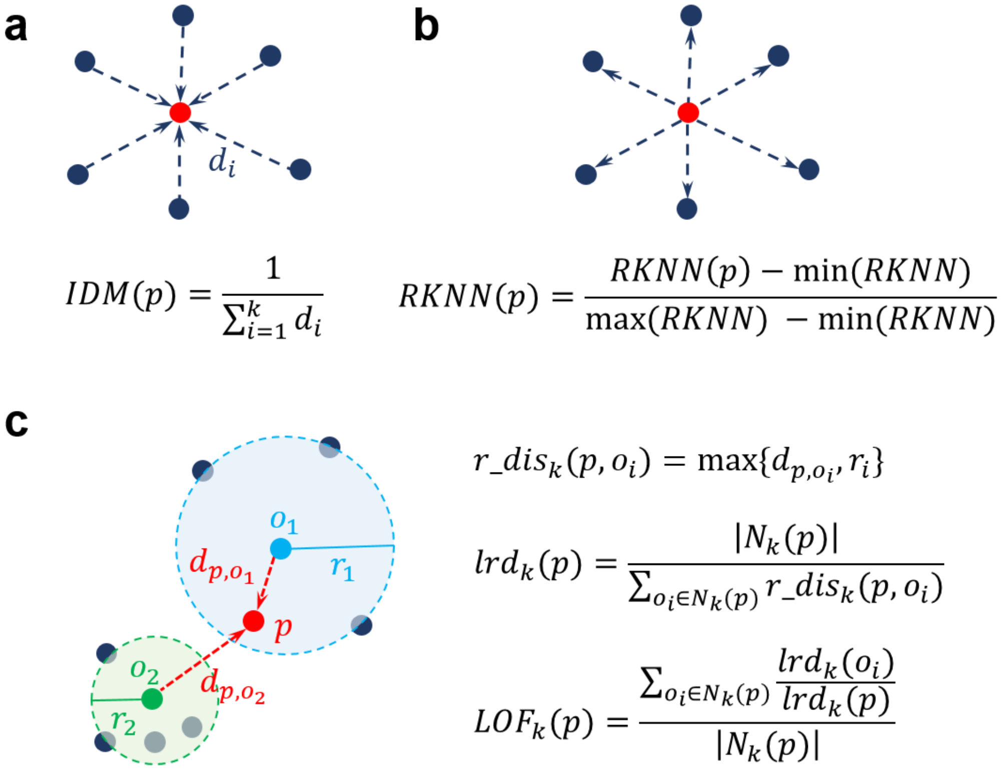

Supplementary Fig. 9. Formulas of three KNN-based methods for noise elimination. (**a**) Inverse Distance Metric (IDM), (**b**) Reverse K-Nearest Neighbors (RKNN), (**c**) Local Outlier Factor (LOF).

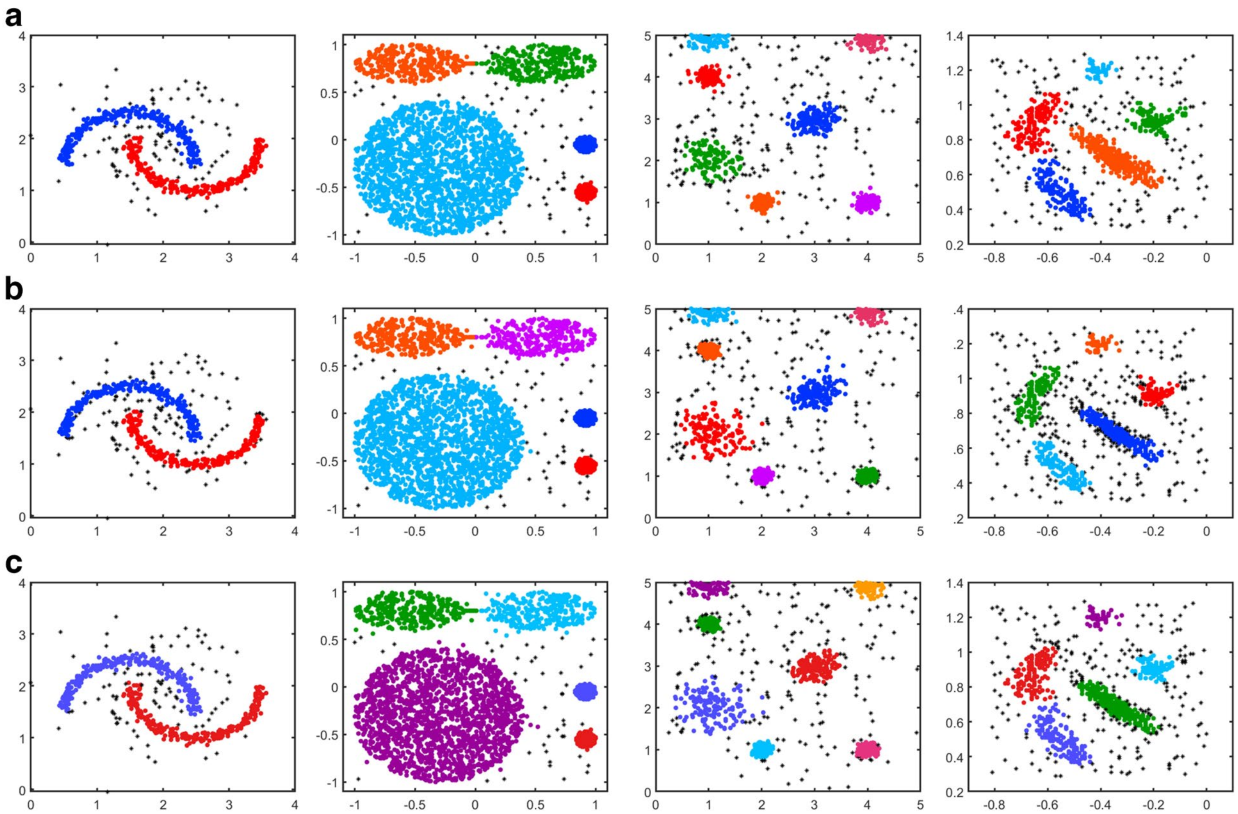

Supplementary Fig. 10. Clustering results of CDC equipped with three noise elimination methods, (a) IDM, (b) RKNN, (c) LOF, on four synthetic datasets (DS10-DS13) with noise respectively.

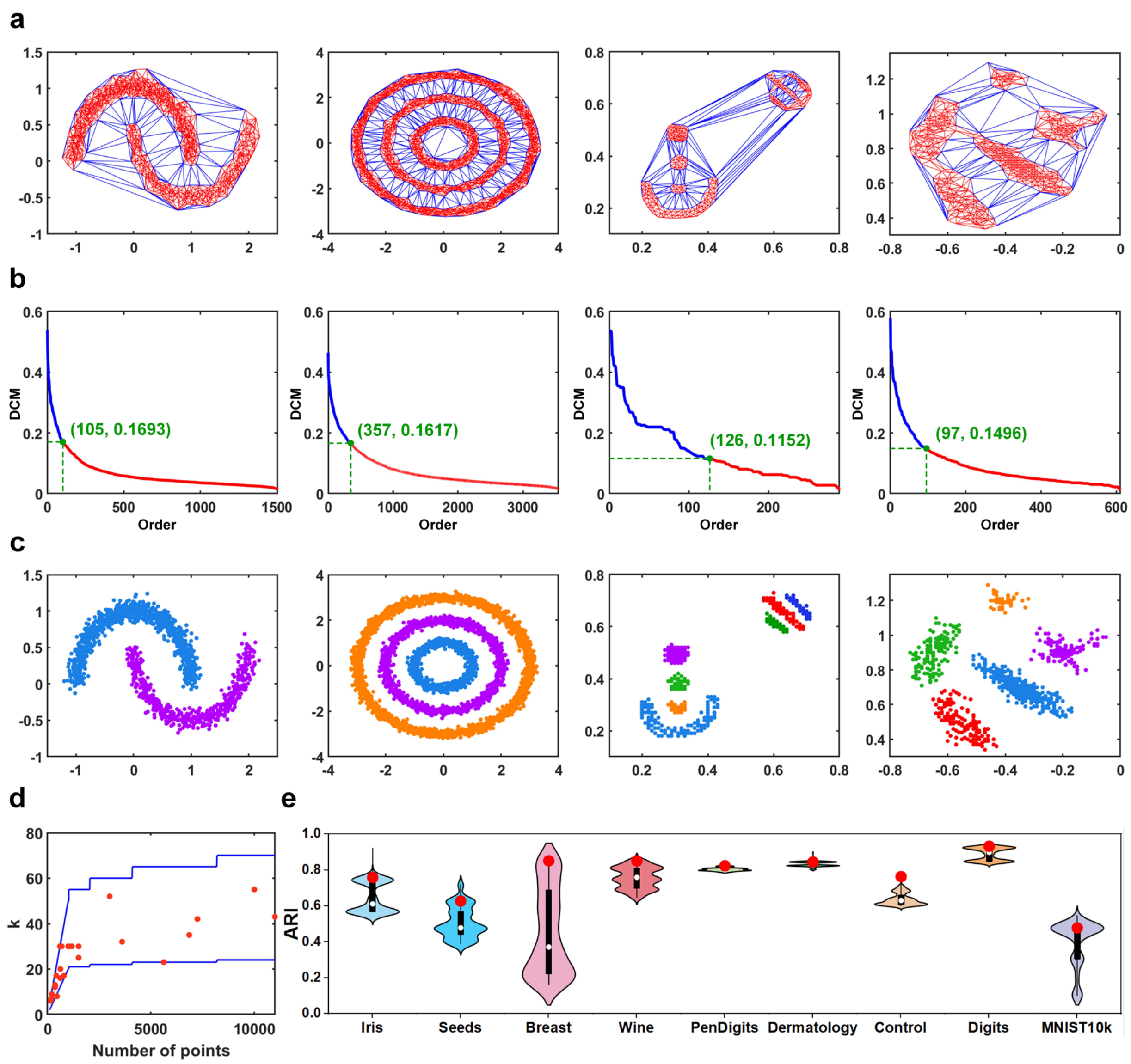

Supplementary Fig. 11. Illustration of T_{DCM} estimation and performance of the parameter adaption methods. (**a**) Triangulations generated by Delaunay triangulation algorithm on four synthetic datasets (DS14-DS17), where the blue and red triangles represent the extracted cross-cluster and intra-cluster triangles respectively. (**b**) Curves formed by the sorted *DCMs* in a descend order, where the estimated numbers of boundary points are 110, 342, 127 and 87, and the determined T_{DCM} are 0.1693, 0.1617, 0.1152 and 0.1496 respectively. (**c**) Clustering results using the estimated T_{DCM}. (**d**) Distribution of the optimal *k*, where the blue lines denote the estimated upper and lower bounds, and the red points represent the optimal *k* on 17 synthetic and 9 real-world datasets. (**e**) ARIs of adaptive CDC (red points) and ARI distributions of the top 200 clustering results with a random parameter setting on nine real-world datasets. Boxes show the median and the 25-75% range, while whiskers refer to the 1.5 times interquartile range.

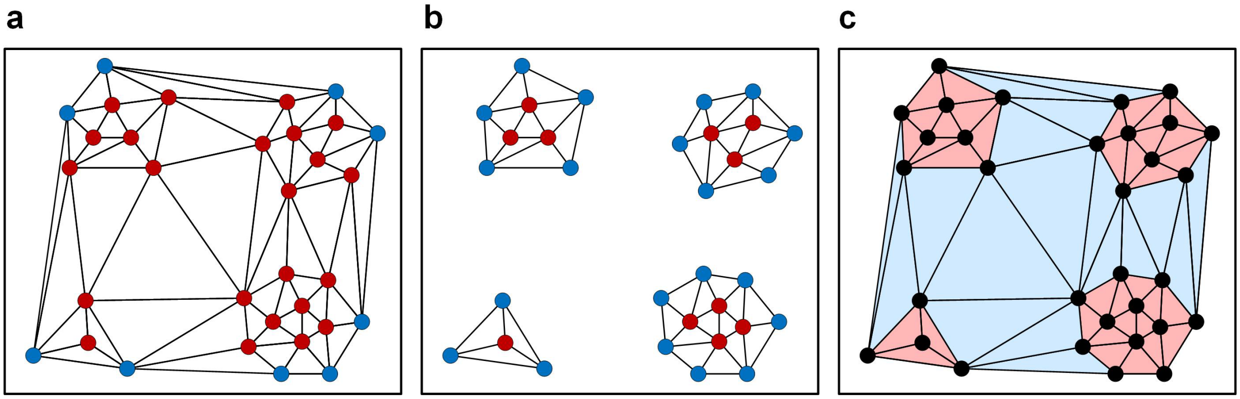

Supplementary Fig. 12. Estimation of the number of boundary points. (**a**) A single network of triangulation generated by all points, where the blue points represent the boundary points and the red ones represent the internal points. (**b**) Ideal triangulation networks of separated clusters with correct boundary points. (**c**) Identification of the cross-cluster triangles (blue) from a whole TIN, and red ones represent intra-cluster triangles.

Supplementary Table 1. Information of the scRNA-seq and CyTOF datasets used in cell type identification.

Dataset	No. of cells	No. of expressed genes	No. of cell populations	Description	Platform
BH	8,569	17,499	14	Human pancreas	inDrop
BM	1,886	14,878	13	Mouse pancreas	inDrop
Muraro	2,122	18,915	9	Human pancreas	CEL-Seq2
Segerstolpe	2,133	22,757	13	Human pancreas	SMART-Seq2
Xin	1,449	33,889	4	Human pancreas	SMARTer
AMB	12,832	42,625	4	Mouse primary visual cortex	SMART-Seq v4
ALM	9,672	42,461	7	Mouse anterior lateral motor cortex	SMART-Seq v4
VISp	14,739	45,768	5	Primary visual cortex	SMART-Seq v4
TM	54,865	19,791	55	Whole Mus musculus	SMART-Seq2
MIHPF	1,093,785	31,053	3/43	Mouse isocortex and hippocampal formation	10X Genomics
WT_R1	7,551	27,998	13	Mouse retinas	10X Genomics
WT_R2	7,695	27,998	13	Mouse retinas	10X Genomics
NdpKO_R1	9,939	27,998	13	Mouse retinas	10X Genomics
NdpKO_R2	7,640	27,998	13	Mouse retinas	10X Genomics
**Dataset**	**No. of cells**	**No. of protein marker dimensions**	**No. of cell populations**	**Description**	**Platform**
Levine	265,627	33	14	Human bone marrow	FlowRepository
Samusik	841,644	40	24	Mouse bone marrow	FlowRepository

Supplementary Table 2. Clustering accuracy reported by the max (mean ±standard deviation) ARIs of 15 algorithms equipped with PCA or UMAP on nine scRNA-seq datasets. The red and blue color represents the highest max and average ARI scores respectively, green color indicates the smallest standard deviation (do not consider CDC-U3, CDC-U4 and CDC-U5), and "/" means the algorithm is not applicable. The rank scores of all algorithms are summarized in the last column (the smaller, the better).

	BH	BM	Muraro	Segerstolpe	Xin	AMB	ALM	VISp	TM	Rank
CDC-U2	**0.953**(**0.918**) ±0.049	**0.968**(**0.963**) **±0.013**	0.924(**0.841**) ±0.147	**0.973**(**0.963**) **±0.006**	0.973(**0.921**) ±0.147	**0.887**(**0.608**) ±0.134	**0.719**(**0.625**) ±0.184	**0.857**(0.485) ±0.241	**0.838**(**0.805**) ±0.055	**1.8**(**1.2**)
CDC-U3	*0.964(0.821) ±0.143*	*0.979(0.878) ±0.062*	*0.930(0.862) ±0.118*	*0.973(0.943) ±0.020*	*0.970(0.902) ±0.125*	*0.678(0.606) ±0.046*	*0.437(0.396) ±0.031*	*0.614(0.545) ±0.047*	*0.833(0.770) ±0.069*	—
CDC-U4	*0.945(0.904) ±0.020*	*0.967(0.961) ±0.009*	*0.924(0.907) ±0.014*	*0.973(0.961) ±0.007*	*0.969(0.943) ±0.008*	*0.396(0.301) ±0.061*	*0.246(0.184) ±0.029*	*0.346(0.267) ±0.064*	*0.800(0.704) ±0.058*	—
CDC-U5	*0.933(0.814) ±0.216*	*0.966(0.920) ±0.155*	*0.923(0.920) ±0.007*	*0.972(0.725) ±0.196*	*0.969(0.932) ±0.033*	*0.344(0.312) ±0.035*	*0.194(0.105) ±0.050*	*0.242(0.148) ±0.083*	*0.616(0.272) ±0.237*	—
Seurat v3	0.944(0.586) ±0.134	0.966(0.577) ±0.150	**0.932**(0.736) ±0.137	0.959(0.622) ±0.153	0.966(0.687) ±0.171	0.691(0.511) ±0.086	0.518(0.364) ±0.114	0.610(0.432) ±0.123	0.806(0.689) ±0.075	6.1(4.8)
monocle3	0.764(0.493) ±0.075	0.683(0.528) ±0.123	0.916(0.756) ±0.115	0.446(0.232) ±0.124	0.592(0.334) ±0.162	0.493(0.353) ±0.063	0.430(0.252) ±0.086	0.439(0.336) ±0.074	0.809(0.575) ±0.160	17.4(10.9)
SC3	0.912(0.463) ±0.174	0.680(0.305) ±0.151	0.932(0.446) ±0.225	0.892(0.365) ±0.201	**0.984**(0.171) ±0.223	0.656(0.506) ±0.155	0.591(0.422) ±0.155	0.583(0.429) ±0.157	0.716(0.564) ±0.146	9.8(10.4)
dropClust	0.639(0.573) ±0.063	0.839(0.699) ±0.128	0.932(0.836) ±0.091	0.936(0.747) ±0.185	0.979(0.799) ±0.171	0.580(0.546) **±0.035**	0.343(0.227) ±0.115	0.457(0.279) ±0.176	0.725(0.723) **±0.001**	12.9(5.6)
MetaCell	0.134(0.075) **±0.020**	0.339(0.200) ±0.057	0.461(0.240) **±0.076**	0.447(0.257) ±0.079	0.273(0.154) **±0.042**	0.400(0.281) ±0.060	0.566(0.443) ±0.081	0.437(0.326) ±0.068	0.064(0.037) ±0.010	21.0(18.1)
SNN-Walktrap-U2	0.475(0.211) ±0.104	0.561(0.227) ±0.101	0.508(0.258) ±0.091	0.560(0.248) ±0.107	0.321(0.144) ±0.066	0.720(0.482) ±0.140	0.611(0.483) ±0.105	0.638(0.427) ±0.110	0.560(0.251) ±0.120	14.6(15.6)
SNN-Walktrap-PCA	0.605(0.498) ±0.050	0.654(0.461) ±0.112	0.736(0.596) ±0.102	0.736(0.486) ±0.174	0.926(0.698) ±0.183	0.726(0.578) ±0.056	0.626(0.494) ±0.042	0.659(0.530) ±0.048	0.771(0.709) ±0.047	11.6(4.2)
SNN-Louvain-U2	0.424(0.203) ±0.101	0.597(0.452) ±0.089	0.445(0.256) ±0.097	0.431(0.243) ±0.105	0.293(0.143) ±0.071	0.606(0.452) ±0.126	0.598(0.491) ±0.109	0.548(0.411) ±0.104	0.423(0.231) ±0.104	18.7(15.0)
SNN-Louvain-PCA	0.593(0.514) ±0.064	0.404(0.225) ±0.100	0.730(0.555) ±0.124	0.718(0.472) ±0.127	0.852(0.564) ±0.165	0.694(0.592) ±0.054	0.576(0.499) ±0.041	0.621(**0.534**) ±0.048	0.731(0.629) ±0.064	14.1(6.4)
Kmeans-U2	0.802(0.375) ±0.176	0.871(0.281) ±0.189	0.823(0.328) ±0.173	0.806(0.315) ±0.151	0.940(0.184) ±0.204	0.668(0.460) ±0.140	0.547(0.397) ±0.139	0.600(0.389) ±0.117	0.731(0.460) ±0.099	13.0(13.9)
Kmeans-PCA	0.924(0.350) ±0.185	0.937(0.308) ±0.209	0.930(0.397) ±0.232	0.965(0.355) ±0.213	0.980(0.273) ±0.241	0.667(0.376) ±0.133	0.568(0.241) ±0.175	0.608(0.271) ±0.190	0.666(0.475) ±0.196	8.0(13.7)
Cmeans-U2	0.854(0.348) ±0.176	0.950(0.274) ±0.190	0.905(0.327) ±0.195	0.910(0.327) ±0.199	0.862(0.173) ±0.190	0.624(0.433) ±0.118	0.526(0.384) ±0.129	0.539(0.377) ±0.103	0.667(0.463) ±0.111	14.2(15.1)
Cmeans-PCA	0.885(0.469) ±0.240	0.907(0.459) ±0.251	0.914(0.475) ±0.196	0.902(0.456) ±0.254	0.961(0.587) ±0.311	0.502(0.237) ±0.124	0.443(0.200) ±0.126	0.496(0.175) ±0.127	0.639(0.368) ±0.120	14.9(11.9)
CLARA-U2	0.866(0.404) ±0.190	0.944(0.287) ±0.218	0.917(0.345) ±0.212	0.968(0.340) ±0.209	0.866(0.225) ±0.177	0.579(0.447) ±0.130	0.568(0.396) ±0.157	0.562(0.417) ±0.140	0.680(0.509) ±0.122	12.1(12.2)
CLARA-PCA	0.895(0.390) ±0.150	0.903(0.356) ±0.149	0.918(0.501) ±0.211	0.940(0.418) ±0.195	0.955(0.252) ±0.189	0.608(0.372) ±0.134	0.434(0.151) ±0.118	/	/	14.8(14.4)
DBSCAN-U2	0.953(0.698) ±0.389	0.964(0.624) ±0.416	0.921(0.614) ±0.400	0.956(0.592) ±0.417	0.966(0.631) ±0.399	0.664(0.228) ±0.139	0.486(0.193) ±0.115	0.619(0.203) ±0.130	0.820(0.497) ±0.231	6.9(9.4)
DBSCAN-PCA	0.445(0.060) ±0.122	0.702(0.083) ±0.171	0.592(0.051) ±0.114	0.526(0.064) ±0.133	0.931(0.081) ±0.226	0.163(0.014) ±0.033	0.135(0.007) ±0.019	0.166(0.006) **±0.018**	0.403(0.036) ±0.078	19.9(22.7)
hclust-U2	0.949(0.437) ±0.305	0.964(0.326) ±0.282	0.923(0.359) ±0.284	0.968(0.369) ±0.286	0.966(0.282) ±0.285	0.737(0.363) ±0.223	0.601(0.285) ±0.198	0.624(0.311) ±0.197	/	5.3(13.4)
hclust-PCA	0.946(0.215) ±0.201	0.964(0.271) ±0.237	0.924(0.272) ±0.255	0.953(0.249) ±0.234	0.964(0.212) ±0.263	0.663(0.171) ±0.189	0.590(0.055) ±0.122	0.592(0.063) ±0.136	/	8.7(19.7)
DIANA-U2	0.935(0.430) ±0.225	0.963(0.281) ±0.208	0.923(0.346) ±0.220	0.968(0.340) ±0.209	0.842(0.202) ±0.207	0.661(0.510) ±0.156	0.580(0.399) ±0.164	0.565(0.428) ±0.137	/	10.7(12.4)
DIANA-PCA	0.865(0.303) ±0.123	0.893(0.445) ±0.194	0.874(0.344) ±0.186	0.940(0.418) ±0.195	0.944(0.294) ±0.270	0.610(0.292) ±0.173	0.007(0.006) **±0.001**	/	/	16.3(15.9)
AGNES-U2	0.948(0.546) ±0.286	0.964(0.423) ±0.311	0.923(0.452) ±0.272	0.968(0.438) ±0.262	0.966(0.360) ±0.331	0.694(0.437) ±0.174	0.601(0.350) ±0.175	/	/	7.7(11.7)
AGNES-PCA	0.937(0.252) ±0.199	0.964(0.337) ±0.226	0.918(0.344) ±0.258	0.933(0.306) ±0.235	0.962(0.272) ±0.288	0.661(0.209) ±0.189	0.482(0.043) ±0.100	/	/	12.6(17.9)

Supplementary Table 3. UCI, handwritten and face image datasets used in the experiment of dimension expansion.

Dataset	No. of samples	No. of dimensions	No. of classes
Iris	150	4	3
Seeds	210	7	3
Breast-Cancer	683	10	2
Wine	178	13	3
PenDigits	10,992	16	10
Dermatology	358	34	6
Control	600	60	6
Digits	5,620	8×8 (64)	10
MNIST10k	10,000	28×28 (784)	10
ORL	400	2240	40

Supplementary Table 4. ACC scores of CDC and other 10 algorithms on nine real-world datasets. The highest ACC score of all clustering algorithms in each dataset is highlighted in bold. The rank scores of all algorithms are summarized in the last column.

	Iris	Seeds	Breast	Wine	PenDigits	Dermatology	Control	Digits	MNIST10k	Rank
K-means	0.8867	0.8905	0.9605	0.9438	0.6616	0.8134	0.5683	0.7975	0.5269	7.3
DBSCAN	0.6733	0.5810	0.7570	0.5956	0.3854	0.5056	0.5500	0.6480	0.1028	11.8
CDP	0.6533	0.8905	0.8199	0.8820	0.5987	0.7437	0.5350	0.3863	0.2956	10.6
AGNES	0.6600	0.6000	0.7672	0.5955	0.3856	0.5042	0.5333	0.2014	0.1027	12.9
MeanShift	0.6667	0.8524	**0.9619**	0.9045	0.7740	0.7939	0.5733	0.8103	0.1873	7.9
Rcut	0.6733	0.5810	0.5766	0.6011	0.5627	0.5014	0.5710	0.5599	0.5794	11.1
Ncut	0.6657	0.6685	0.8635	0.6685	0.7332	0.7019	**0.7367**	0.8114	0.5103	8.7
densityCut	0.6667	0.8952	0.7980	0.8933	0.8089	0.8212	0.6667	0.9084	0.7215	6.8
RCC	0.7733	0.7846	0.3104	0.9438	0.8094	0.8631	0.6667	0.9052	0.5865	7.2
GCSED	0.5333	0.8333	0.9385	0.9551	0.7273	0.8301	0.6433	0.9382	0.6512	7.6
CDC-U2	**0.9667**	0.8952	0.9546	0.9663	**0.8389**	0.8659	0.6833	**0.9495**	0.7452	2.8
CDC-U3	**0.9667**	0.9048	0.9546	0.9663	0.8218	0.8659	0.6867	0.9472	0.7424	2.9
CDC-U4	**0.9667**	**0.9095**	0.9546	**0.9719**	0.8312	**0.8743**	0.7033	0.9452	0.7707	**2.0**
CDC-U5	/	0.9048	0.9561	0.9607	0.8281	0.8659	0.7067	0.9484	**0.7505**	2.3

Supplementary Table 5. NMI scores of CDC and other 10 algorithms on nine real-world datasets. The highest NMI score of all clustering algorithms in each dataset is highlighted in bold. The rank scores of all algorithms are summarized in the last column.

	Iris	Seeds	Breast	Wine	PenDigits	Dermatology	Control	Digits	MNIST10k	Rank
K-means	0.7419	0.6743	0.7478	0.8155	0.6778	0.8467	0.7558	0.6826	0.4540	8.0
DBSCAN	0.7337	0.5115	0.4230	0.4945	0.5513	0.6175	0.6437	0.6650	0.1035	11.8
CDP	0.7107	0.6797	0.3866	0.7104	0.6867	0.7509	0.6577	0.4025	0.2033	11.0
AGNES	0.7201	0.5165	0.4230	0.5026	0.5506	0.4529	0.6580	0.1641	0.0877	12.4
MeanShift	0.7337	0.7142	0.7572	0.7729	0.8053	0.8208	0.7444	0.7844	0.2000	7.7
Rcut	0.7235	0.4486	0.5568	0.5114	0.5445	0.7509	0.6577	0.5392	0.5630	11.1
Ncut	0.7225	0.7150	**0.7830**	0.7350	0.7354	0.8548	0.7629	0.6830	0.5317	7.1
densityCut	0.7337	0.6797	0.5400	0.7262	0.8341	0.8507	0.7936	0.8367	0.6337	7.2
RCC	0.7067	0.6602	0.2922	0.8662	0.8667	0.9301	**0.8520**	0.9259	0.7609	6.6
GCSED	0.6478	0.6466	0.7176	0.8529	0.7764	0.8291	0.7178	0.9265	0.7042	8.4
CDC-U2	**0.8851**	0.7190	0.7801	0.8759	**0.8668**	**0.9344**	**0.8520**	0.9425	0.7973	**1.9**
CDC-U3	**0.8851**	0.7529	0.7195	0.8759	0.8656	**0.9344**	0.8181	0.9394	0.7946	2.9
CDC-U4	**0.8851**	**0.7570**	0.7195	**0.8926**	0.8613	**0.9344**	0.8060	0.9392	0.7981	2.9
CDC-U5	/	0.7239	0.7801	0.8609	0.8661	**0.9344**	0.8067	**0.9430**	**0.8074**	2.5

Supplementary Table 6. Sensitivity analysis of parameters in CDC using stratified sampling and random perturbation on six synthetic datasets. The blue shade represents the sensitivity levels, and the darker color indicates the stronger sensitivity.

Parameter	Perturbation	DS4	DS5	DS6	DS7	DS8	DS9
	1	0.0228	0.0308	0.0361	0.0719	0.0112	0.0091
	5	0.0595	0.0609	0.0409	0.0988	0.0374	0.0179
**k**	10	0.0909	0.1005	0.0789	0.1254	0.0639	0.0315
	15	0.1291	0.1371	0.1038	0.1433	0.0856	0.0442
	20	0.1616	0.1502	0.1404	0.1529	0.1043	0.0637
	0.01	0.0580	0.1198	0.0844	0.2403	0.0916	0.0704
	0.05	0.1836	0.2577	0.1888	0.4276	0.5224	0.2525
**T_{DCM}**	0.10	0.2622	0.3809	0.3209	0.6034	0.8385	0.3629
	0.15	0.3921	0.6968	0.4342	0.9683	0.9285	0.4495
	0.20	0.5428	0.7812	0.5021	0.9878	0.9813	0.5644
	0.01	0.0282	0.0385	0.0427	0.0918	0.0242	0.0477
	0.05	0.0982	0.1188	0.0703	0.1866	0.1342	0.0861
**ratio**	0.10	0.1159	0.1956	0.1342	0.2392	0.2671	0.1363
	0.15	0.1727	0.2344	0.2137	0.3020	0.3321	0.1500
	0.20	0.2078	0.2858	0.2686	0.3873	0.3662	0.2322

Sensitivity levels

0 *Insensitive* 0.25 *Mild sensitive* 0.50 *Sensitive* 0.75 *Hypersensitive* 1.00

DCM ranges from 0 to 1 theoretically, but most of *DCMs* commonly lie in a small range. A slight adjustment of T_{DCM} may cause many points to be converted from boundary to internal points or vice versa. This conversion affects the recognition of boundary points and their binding force for internal points, which in turn influences the clustering quality.

In contrast, *ratio* is distributed in [0, 1] completely and all points locate in the range of *ratio* uniformly. A decrease of 0.2 in $ratio$ means that 20% points change from internal points to boundary points, which has slighter effect on the clustering result than T_{DCM} with the same variation. Thus, *ratio* is a less sensitive parameter to CDC.

Supplementary Table 7. Parameter settings of CDC. To be noted, the UMAP (uwot R package) results with the same parameters are slightly different in Windows and macOS machines. The parameter settings for UCI benchmarks can reproduce the same evaluation metrics in a Windows machine.

Parameter settings on 2D synthetic datasets without noise				
DS1	DS2	DS3	DS4	DS5
k = 30, ratio = 0.70	k = 8, ratio = 0.67	k = 11, ratio = 0.92	k = 17, ratio = 0.77	k = 10, ratio = 0.70
DS6	DS7	DS8	DS9	DS14
k = 30, ratio = 0.80	k = 30, ratio = 0.80	k = 52, ratio = 0.80	k = 30, ratio = 0.95	k = 30, ratio = 0.90
DS15	DS16	DS17		
k = 30, ratio = 0.90	k = 9, ratio = 0.60	k = 20, ratio = 0.70		

Parameter settings on 2D synthetic datasets with noise (*T here is noise threshold for the noise elimination methods)			
	IDM	RKNN	LOF
DS10	k = 20, T = 0.30, ratio = 0.90	k = 20, T = 0.38, ratio = 0.80	k = 20, T = 0. 06, ratio = 0.90
DS11	k = 20, T = 0.53, ratio = 0.90	k = 30, T = 0.20, ratio = 0.90	k = 30, T = 0. 09, ratio = 0.90
DS12	k = 30, T = 0.10, ratio = 0.90	k = 30, T = 0.35, ratio = 0.90	k = 30, T = 0. 05, ratio = 0.90
DS13	k = 30, T = 0.50, ratio = 0.70	k = 30, T = 0.35, ratio = 0.80	k = 25, T = 0. 10, ratio = 0.60

Parameter settings on speaker corpuses	
ELSDSR	MSLT
n_components = 2, k = 5, ratio = 0.70	n_components = 2, k = 7, ratio = 0.53

Parameter settings on UCI benchmarks (for obtaining the highest ARI score)

**Dataset**	**Parameter Setting**	**Evaluation Metric**
Iris	n_neighbors = 25, n_components = 2, k = 8, ratio = 0.75	ARI: 0.911, ACC: 0.947, NMI: 0.875
	n_neighbors = 30, n_components = 3, k = 6, ratio = 0.90	ARI: 0.904, ACC: 0.967, NMI: 0.885
	n_neighbors = 25, n_components = 4, k = 11, ratio = 0.90	ARI: 0.904, ACC: 0.967, NMI: 0.885
	/	/
Seeds	n_neighbors = 30, n_components = 2, k = 10, ratio = 0.88	ARI: 0.724, ACC: 0.895, NMI: 0.719
	n_neighbors = 15, n_components = 3, k = 10, ratio = 0.93	ARI: 0.748, ACC: 0.905, NMI: 0.753
	n_neighbors = 30, n_components = 4, k = 6, ratio = 0.88	ARI: 0.759, ACC: 0.910, NMI: 0.757
	n_neighbors = 40, n_components = 5, k = 7, ratio = 0.82	ARI: 0.744, ACC: 0.905, NMI: 0.710
Breast-Cancer	n_neighbors = 25, n_components = 2, k = 8, ratio = 0.975	ARI: 0.849, ACC: 0.955, NMI: 0.780
	n_neighbors = 50, n_components = 3, k = 5, ratio = 0.95	ARI: 0.825, ACC: 0.955, NMI: 0.720
	n_neighbors = 50, n_components = 4, k = 15, ratio = 0.95	ARI: 0.825, ACC: 0.955, NMI: 0.720
	n_neighbors = 25, n_components = 5, k = 11, ratio = 0.95	ARI: 0.850, ACC: 0.956, NMI: 0.780
Wine	n_neighbors = 40, n_components = 2, k = 8, ratio = 0.88	ARI: 0.898, ACC: 0.966, NMI: 0.866
	n_neighbors = 45, n_components = 3, k = 15, ratio = 0.95	ARI: 0.897, ACC: 0.966, NMI: 0.876
	n_neighbors = 15, n_components = 4, k = 17, ratio = 0.94	ARI: 0.915, ACC: 0.972, NMI: 0.893
	n_neighbors = 15, n_components = 5, k = 12, ratio = 0.77	ARI: 0.881, ACC: 0.955, NMI: 0.858
PenDigits	n_neighbors = 20, n_components = 2, k = 20, ratio = 0.95	ARI: 0.823, ACC: 0.822, NMI: 0.867
	n_neighbors = 10, n_components = 3, k = 13, ratio = 0.95	ARI: 0.824, ACC: 0.822, NMI: 0.867
	n_neighbors = 10, n_components = 4, k = 20, ratio = 0.95	ARI: 0.819, ACC: 0.817, NMI: 0.861
	n_neighbors = 20, n_components = 5, k = 10, ratio = 0.95	ARI: 0.824, ACC: 0.822, NMI: 0.866
Dermatology	n_neighbors = 25, n_components = 2, k = 15, ratio = 0.90	ARI: 0.870, ACC: 0.866, NMI: 0.934
	n_neighbors = 25, n_components = 3, k = 10, ratio = 0.95	ARI: 0.870, ACC: 0.866, NMI: 0.934
	n_neighbors = 50, n_components = 4, k = 10, ratio = 0.85	ARI: 0.871, ACC: 0.874, NMI: 0.928
	n_neighbors = 15, n_components = 5, k = 10, ratio = 0.95	ARI: 0.870, ACC: 0.866, NMI: 0.934
Control	n_neighbors = 25, n_components = 2, k = 12, ratio = 0.99	ARI: 0.682, ACC: 0.667, NMI: 0.852
	n_neighbors = 25, n_components = 3, k = 27, ratio = 0.75	ARI: 0.631, ACC: 0.588, NMI: 0.818
	n_neighbors = 20, n_components = 4, k = 10, ratio = 0.85	ARI: 0.632, ACC: 0.703, NMI: 0.764

	n_neighbors = 10, n_components = 5, k = 15, ratio = 0.78	ARI: 0.630, ACC: 0.707, NMI: 0.764
Digits	n_neighbors = 30, n_components = 2, k = 16, ratio = 0.95	ARI: 0.937, ACC: 0.949, NMI: 0.943
	n_neighbors = 40, n_components = 3, k = 26, ratio = 0.95	ARI: 0.935, ACC: 0.947, NMI: 0.939
	n_neighbors = 35, n_components = 4, k = 10, ratio = 0.94	ARI: 0.934, ACC: 0.944, NMI: 0.939
	n_neighbors = 40, n_components = 5, k = 19, ratio = 0.95	ARI: 0.937, ACC: 0.948, NMI: 0.943
MNIST10k	n_neighbors = 15, n_components = 2, k = 30, ratio = 0.85	ARI: 0.700, ACC: 0.743, NMI: 0.797
	n_neighbors = 10, n_components = 3, k = 35, ratio = 0.86	ARI: 0.697, ACC: 0.740, NMI: 0.795
	n_neighbors = 20, n_components = 4, k = 23, ratio = 0.87	ARI: 0.701, ACC: 0.745, NMI: 0.798
	n_neighbors = 10, n_components = 5, k = 12, ratio = 0.94	ARI: 0.706, ACC: 0.751, NMI: 0.807
