# Supplementary material for: Clustering by measuring local direction centrality for data with heterogeneous density and weak connectivity
Source: Nat Commun. 2022 Sep 16;13:5455. doi: 10.1038/s41467-022-33136-9 (PMC9481560; doi:10.1038/s41467-022-33136-9)
Supplement: Supplementary file 2 — Reporting Summary [file 41467_2022_33136_MOESM2_ESM.pdf]

nature research

Corresponding author(s): Zhipeng Gui

Last updated by author(s): Aug 29, 2022

Reporting Summary

Nature Research wishes to improve the reproducibility of the work that we publish. This form provides structure for consistency and transparency in reporting. For further information on Nature Research policies, see our Editorial Policies and the Editorial Policy Checklist.

Statistics

For all statistical analyses, confirm that the following items are present in the figure legend, table legend, main text, or Methods section.

n/a	Confirmed	
☐	☒	The exact sample size (n) for each experimental group/condition, given as a discrete number and unit of measurement
☐	☒	A statement on whether measurements were taken from distinct samples or whether the same sample was measured repeatedly
☒	☐	The statistical test(s) used AND whether they are one- or two-sided<br>*Only common tests should be described solely by name; describe more complex techniques in the Methods section.*
☒	☐	A description of all covariates tested
☐	☒	A description of any assumptions or corrections, such as tests of normality and adjustment for multiple comparisons
☐	☒	A full description of the statistical parameters including central tendency (e.g. means) or other basic estimates (e.g. regression coefficient) AND variation (e.g. standard deviation) or associated estimates of uncertainty (e.g. confidence intervals)
☒	☐	For null hypothesis testing, the test statistic (e.g. F, t, r) with confidence intervals, effect sizes, degrees of freedom and P value noted<br>*Give P values as exact values whenever suitable.*
☒	☐	For Bayesian analysis, information on the choice of priors and Markov chain Monte Carlo settings
☒	☐	For hierarchical and complex designs, identification of the appropriate level for tests and full reporting of outcomes
☒	☐	Estimates of effect sizes (e.g. Cohen's d, Pearson's r), indicating how they were calculated

Our web collection on statistics for biologists contains articles on many of the points above.

Software and code

Policy information about availability of computer code

Data collection	R (version 4.1.0), MATLAB (version R2020b) and Python (version 3.9.1) were included for data collection in this research.
Data analysis	R package Seurat (version 3.2.2), monocle3 (version 0.2.1), sc3 (version 1.14.0), dropClust (version 2.1.0), scran (version 1.14.6), igraph (version 1.2.6), clusterX, DensVM, flowClust, flowMeans, flowMerge, flowPeaks, flowSOM, immunoClust, Rclusterpp, SamSPECTRAL (version 4.0.3), ACCENSE (version 0.5.1), MATLAB function K-means, DBSCAN, MeanShift (version R2020b) were included for data analysis in this research.

For manuscripts utilizing custom algorithms or software that are central to the research but not yet described in published literature, software must be made available to editors and reviewers. We strongly encourage code deposition in a community repository (e.g. GitHub). See the Nature Research guidelines for submitting code & software for further information.

Data

Policy information about availability of data

All manuscripts must include a data availability statement. This statement should provide the following information, where applicable:

- Accession codes, unique identifiers, or web links for publicly available datasets
- A list of figures that have associated raw data
- A description of any restrictions on data availability

The synthetic datasets used in this study have been deposited at https://github.com/ZPGuiGroupWhu/ClusteringDirectionCentrality/tree/master/Toolkit/Synthetic%20Data%20Analysis/SyntheticDatasets. The scRNA-seq, CyTOF, corpus and other real-world datasets used in this study are available publicly: BH, BM, Muraro, Segerstolpe, Xin, AMB and TM (https://zenodo.org/record/2877646#.YjBPGXpByUl), ALM and VISp (https://portal.brain-map.org/atlases-and-data/rnaseq/mouse-v1-and-alm-smart-seq), WT_R1, WT_R2, NdpKO_R1 and NdpKo_R2 (GSE125708), MIHPF (https://portal.brain-map.org/atlases-and-data/rnaseq/mouse-whole-cortex-and-hippocampus-10x), Levine and Samusik (FlowRepository: FR-FCM-ZZPH), ELSDSR (http://www2.imm.dtu.dk/~lfen/elsdsr/), MSLT (https://

www.microsoft.com/en-us/download/details.aspx?id=55951), Iris (http://archive.ics.uci.edu/ml/datasets/Iris), Seeds (http://archive.ics.uci.edu/ml/datasets/seeds), Breast-Cancer (http://archive.ics.uci.edu/ml/datasets/Breast+Cancer+Wisconsin+%28Original%29), Wine (http://archive.ics.uci.edu/ml/datasets/Wine), PenDigits (http://archive.ics.uci.edu/ml/datasets/Pen-Based+Recognition+of+Handwritten+Digits), Dermatology (http://archive.ics.uci.edu/ml/datasets/Dermatology), Control (http://archive.ics.uci.edu/ml/datasets/Synthetic+Control+Chart+Time+Series), Digits (https://archive.ics.uci.edu/ml/datasets/Optical+Recognition+of+Handwritten+Digits), MNIST10k (http://yann.lecun.com/exdb/mnist/), ORL face dataset (ftp://ftp.uk.research.att.com:pub/data/att_faces.tar.Z).

Field-specific reporting

Please select the one below that is the best fit for your research. If you are not sure, read the appropriate sections before making your selection.

[x] Life sciences   [ ] Behavioural & social sciences   [ ] Ecological, evolutionary & environmental sciences

For a reference copy of the document with all sections, see nature.com/documents/nr-reporting-summary-flat.pdf

Life sciences study design

All studies must disclose on these points even when the disclosure is negative.

Sample size	The validity of our algorithm is demonstrated by detecting complex structured clusters in 17 challenging synthetic datasets, identifying cell types from 14 scRNA-seq and two CyTOF datasets, recognizing speakers on two voice corpuses, and testifying on eight UCI, one handwritten image, one face image benchmarks.
Data exclusions	For single cell analysis, cells were filtered that have the unique feature counts over 2,500 or less than 200, and have >5% mitochondrial counts.
Replication	All experimental results can be reproduced using the toolkit and parameter settings we provided.
Randomization	Not applicable as this is not a case-control experiment and all data analyses were performed on the entire datasets.
Blinding	Given this is not a case-control experiment then blinding of the data is not appropriate

Reporting for specific materials, systems and methods

We require information from authors about some types of materials, experimental systems and methods used in many studies. Here, indicate whether each material, system or method listed is relevant to your study. If you are not sure if a list item applies to your research, read the appropriate section before selecting a response.

Materials & experimental systems

n/a	Involved in the study	
[x]	[ ]	Antibodies
[x]	[ ]	Eukaryotic cell lines
[x]	[ ]	Palaeontology and archaeology
[x]	[ ]	Animals and other organisms
[x]	[ ]	Human research participants
[x]	[ ]	Clinical data
[x]	[ ]	Dual use research of concern

Methods

n/a	Involved in the study	
[x]	[ ]	ChIP-seq
[x]	[ ]	Flow cytometry
[x]	[ ]	MRI-based neuroimaging
